# Supplementary figures and images for: Resident microbial communities inhibit growth and antibiotic-resistance evolution of Escherichia coli in human gut microbiome samples
Source: PLoS Biol. 2020 Apr 20;18(4):e3000465. doi: 10.1371/journal.pbio.3000465 (PMC7192512; doi:10.1371/journal.pbio.3000465)

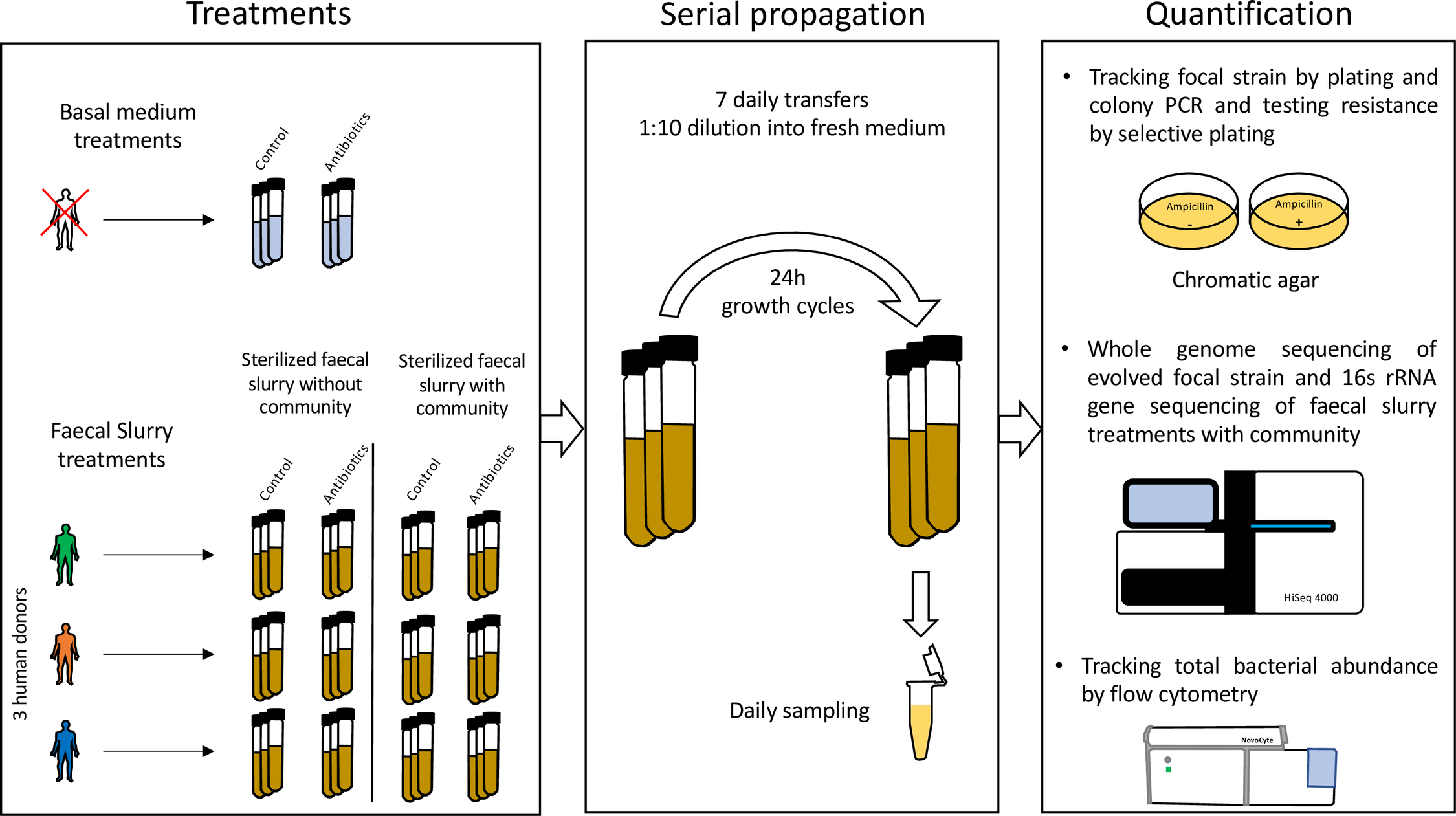

Supplement: S1 Fig — Treatments consisted of basal medium only, basal medium supplemented with sterilised faecal slurry (without the resident microbial community) from one of three human donors, or basal medium supplemented with sterilised faecal slurry to which the resident microbial community had been reintroduced (with community). After inoculation, all treatments were incubated for 2 h at 37°C, before 7 μg/ml ampicillin was added in the antibiotic treatment. Every 24 h, we sampled each microcosm and transferred an aliquot to fresh medium (either basal medium or sterilised faecal slurry) with or without antibiotics. We serially diluted each sample and spread it on chromatic agar plates with or without antibiotics to quantify focal-strain abundance (verified by colony PCR) and to screen for resistance. We sequenced focal-strain isolates from the final time point and investigated community composition by 16S rRNA gene amplicon sequencing. We monitored total bacterial abundance in the community treatments by flow cytometry. (TIF) [file pbio.3000465.s001.tif]

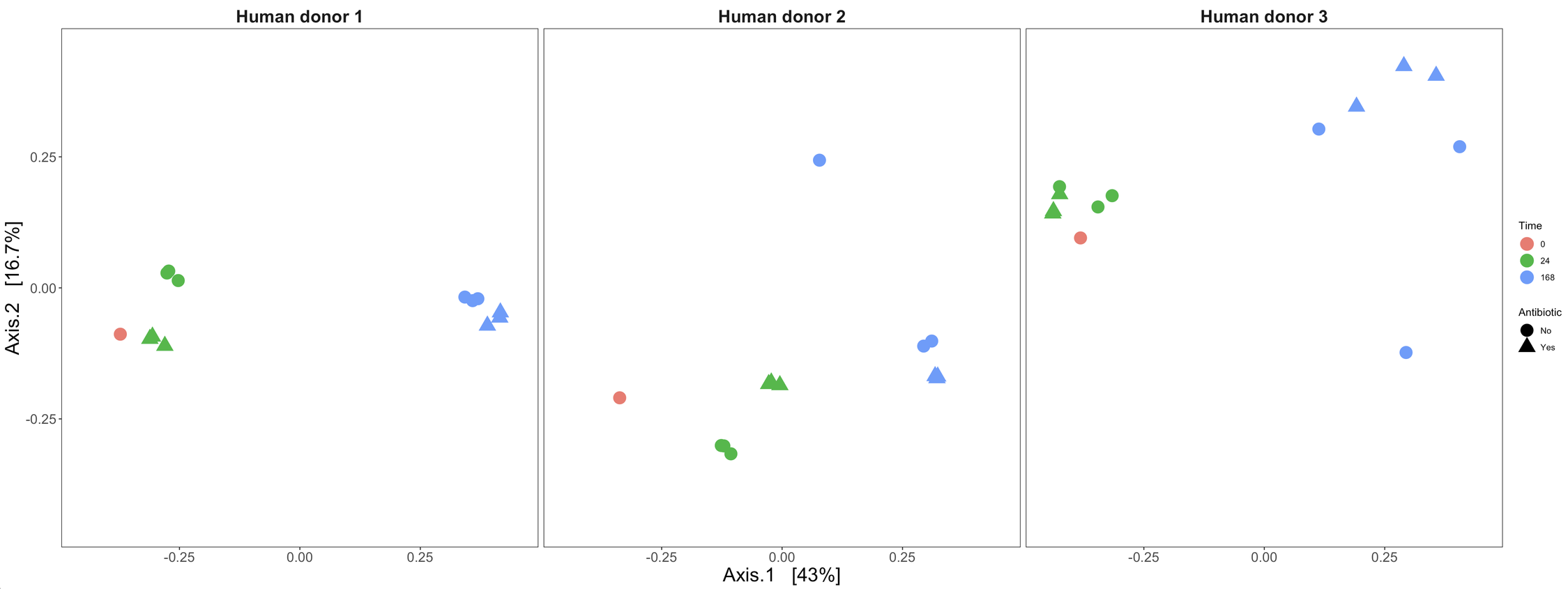

Supplement: S2 Fig — Each panel shows samples from a single human donor, with the same axes used in each panel. Points show the initial sample (0 h) and microcosms from 24 h and 168 h with and without antibiotics (legend at right). Similarities between communities were calculated by Bray-Curtis distance and plotted using principal coordinate analysis (see Material and methods). Data are deposited in the European Nucleotide Archive under the study accession number PRJEB33429. (TIF) [file pbio.3000465.s002.tif]

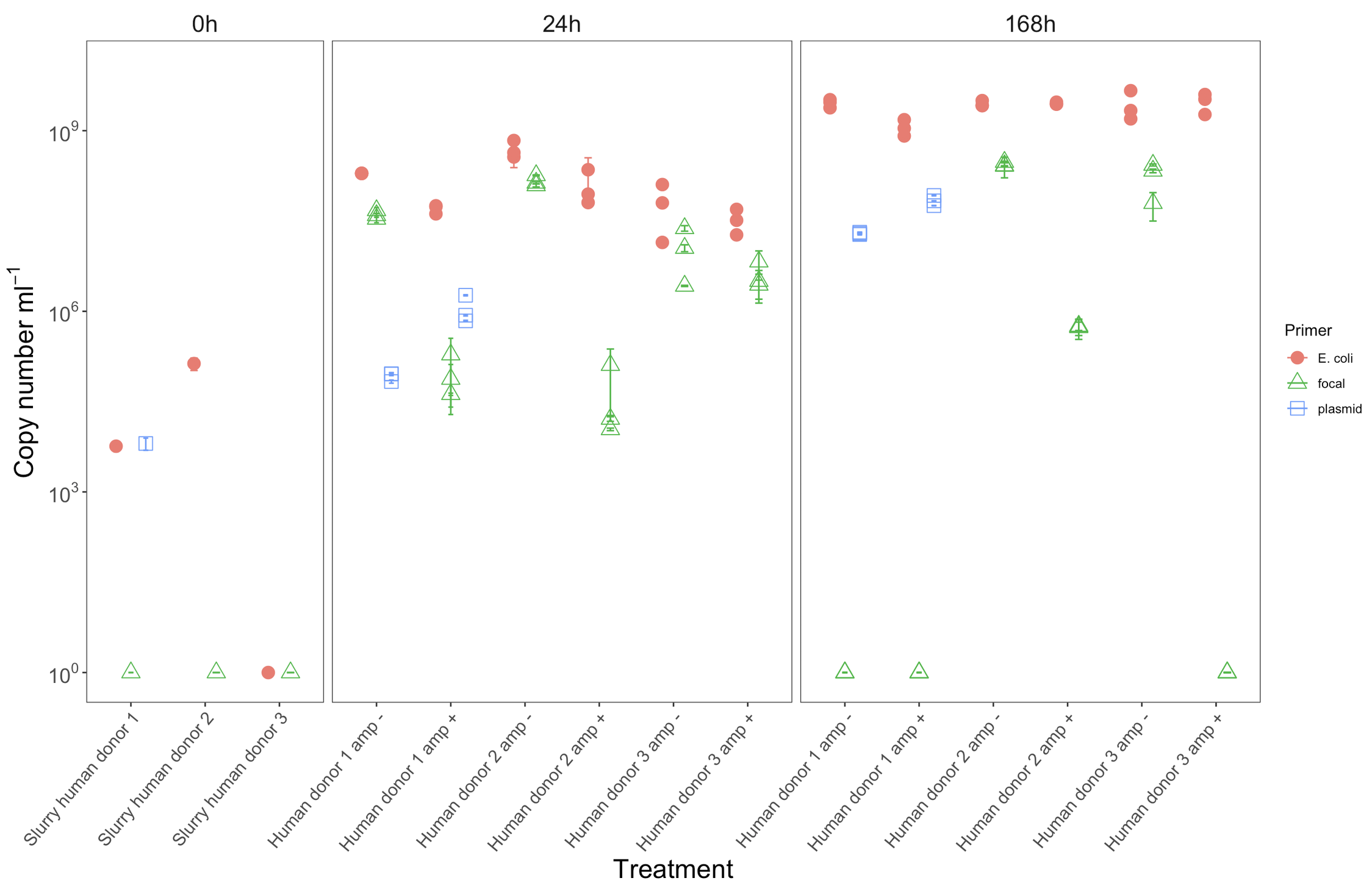

Supplement: S3 Fig — Each panel shows the copy number of sequences detected with primers specific for the focal strain, total E. coli, and the resistance plasmid (see legend; further details of primers in S1 Methods) at time point 0 h (left panel), 24 h (middle panel), and 168 h (right panel). Each point shows the mean of three technical replicates. Reactions in which no amplification was detected are shown at 100. We expect plasmid copy number to reflect the abundance of plasmid donor cells, because coverage analysis of whole-genome sequencing data indicated a copy number per cell of approximately 1. For the focal strain and total E. coli, the copy number of sequences does not necessarily reflect the total number of cells of each type, but changes in strain abundance over time would nevertheless be expected to result in strongly correlated changes in sequence copy numbers over time. Data are deposited in the Dryad repository: https://doi.org/10.5061/dryad.t1g1jwszq [40]. qPCR, quantitative PCR. (TIF) [file pbio.3000465.s003.tif]

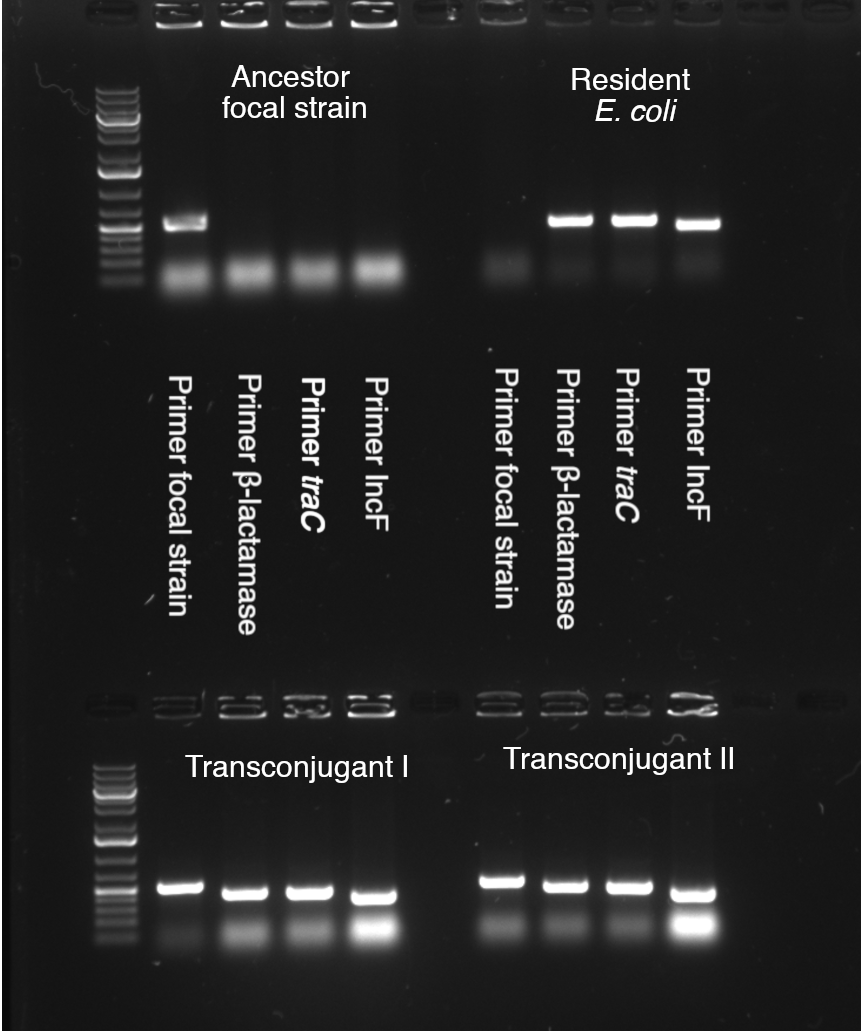

Supplement: S4 Fig — We used these primer sets to verify plasmid uptake of the transconjugants. Primers are given in the main text in the Material and methods section. (TIF) [file pbio.3000465.s004.tif]

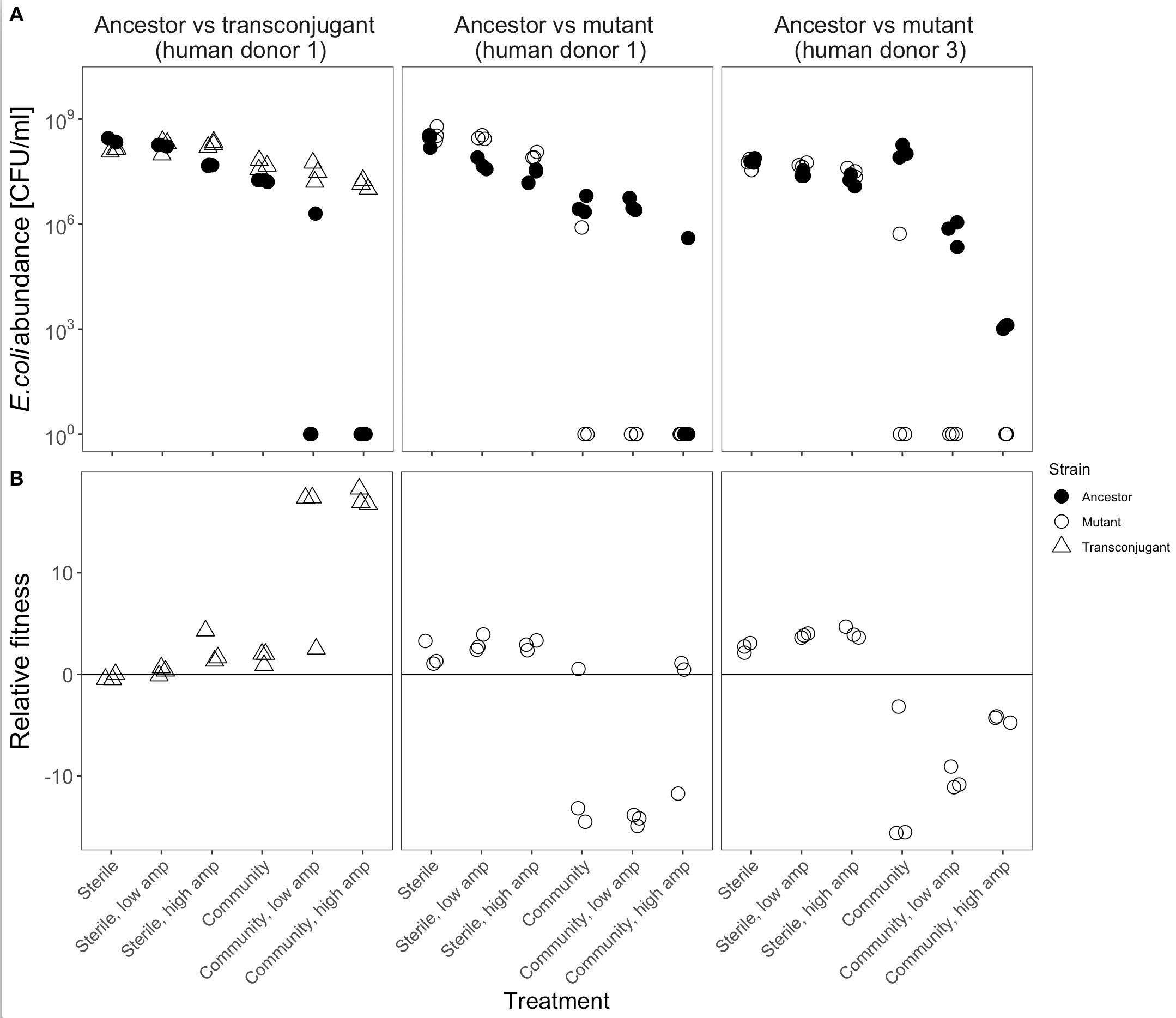

Supplement: S5 Fig — (A) Final cell densities of competing strains (see legend; Transconjugant is a transconjugant of the focal strain carrying the plasmid from human donor 1, in the left panel; Mutant is an evolved isolate with increased ampicillin resistance from the community-free treatments with slurry from human donor 1, in the middle panel, or human donor 3, in the right panel; Ancestor is the respective ancestral focal strain). Data are shown after 24 h of competition in sterile slurry or community treatments, with and without low or high concentrations of ampicillin (x-axis). (B) Fitness of the transconjugant or mutant relative to the ancestor, calculated as the difference of their Malthusian growth rate in the same experiment. In both panels, the three points show three replicates of the experiment. Data are deposited in the Dryad repository: https://doi.org/10.5061/dryad.t1g1jwszq [40]. MIC, minimal inhibitory concentration. (TIF) [file pbio.3000465.s005.tif]

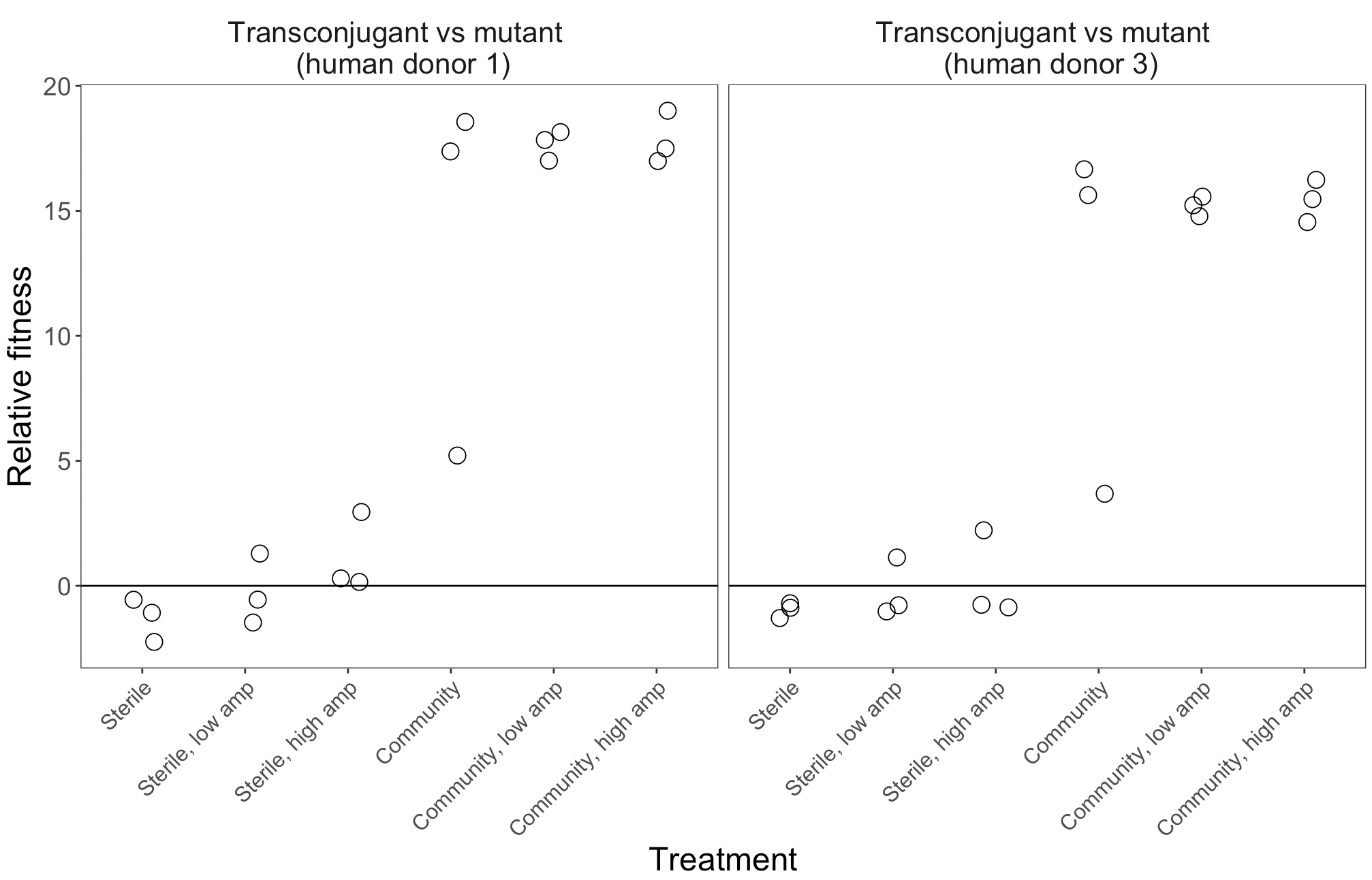

Supplement: S6 Fig — Competitive fitness of transconjugants (carrying the plasmid from resident E. coli of human donor 1) relative to evolved isolates (from community-free treatments with faecal slurry from human donor 1, left, and human donor 3, right). In each panel, relative fitness of the transconjugant strain is shown as the difference in Malthusian growth rates compared with the respective evolved isolate (see S1 Methods). Competitions were done in sterile faecal slurry or the presence of the resident microbial community and with no, low, or high ampicillin concentrations (x-axis). Each point shows a different replicate. Data are deposited in the Dryad repository: https://doi.org/10.5061/dryad.t1g1jwszq [40]. (TIF) [file pbio.3000465.s006.tif]

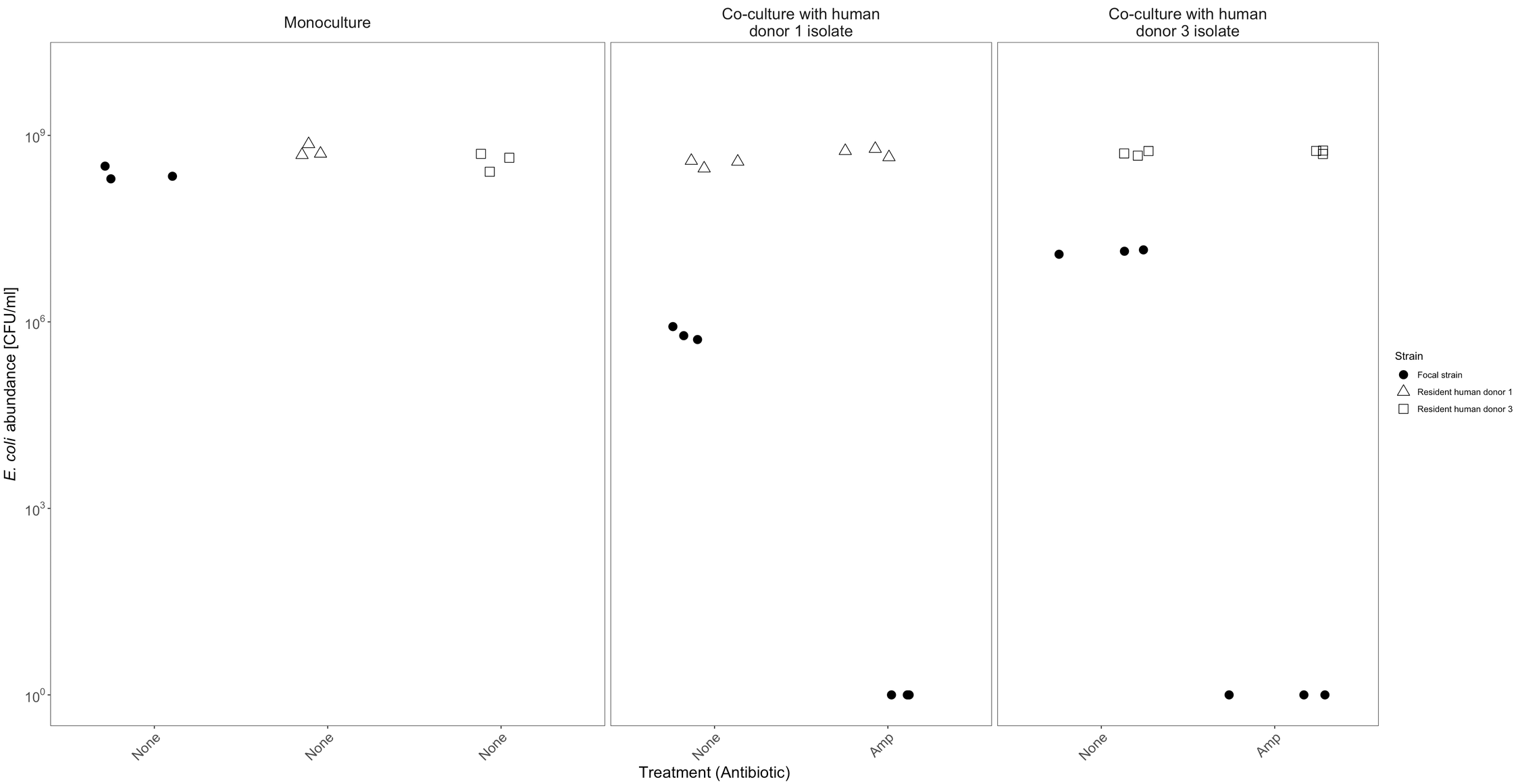

Supplement: S7 Fig — Abundance of the focal E. coli strain and resident E. coli strains isolated from human donors 1 and 3 (see legend) in monoculture (left) and in coculture (right). Each strain was grown in monoculture in the absence of antibiotics, and each coculture combination was grown in the presence and absence of ampicillin (x-axis). Each point shows a different replicate. Data are deposited in the Dryad repository: https://doi.org/10.5061/dryad.t1g1jwszq [40]. (TIF) [file pbio.3000465.s007.tif]

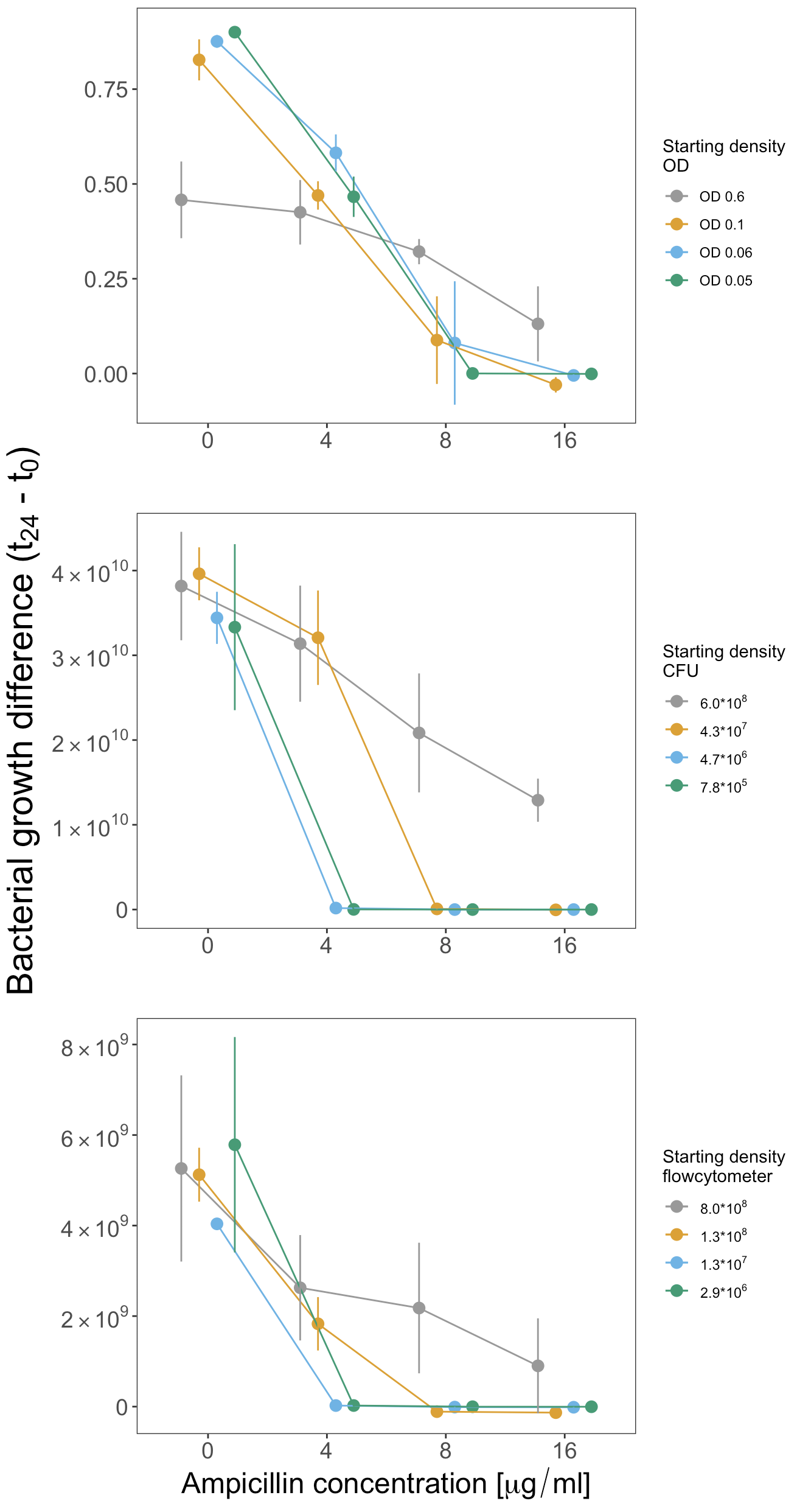

Supplement: S8 Fig — Changes in bacterial abundance over 24 h are shown using three different quantification methods (OD, top panel; plating and CFU counting, middle panel; flow cytometry, bottom panel). In each panel, the change in abundance is shown for four starting densities (see legend) and at four antibiotic concentrations. In each panel, the change between 0 h and 24 h is shown (in OD in the top panel, in CFU/ml in the middle panel, and in recorded events/ml in the bottom panel). Each point shows the mean of three replicates; error bars show 1 SD. Data are deposited in the Dryad repository: https://doi.org/10.5061/dryad.t1g1jwszq [40]. CFU, colony-forming units; OD, optical density. (TIF) [file pbio.3000465.s008.tif]
