# Supplementary material for: Resident microbial communities inhibit growth and antibiotic-resistance evolution of Escherichia coli in human gut microbiome samples
Source: PLoS Biol. 2020 Apr 20;18(4):e3000465. doi: 10.1371/journal.pbio.3000465 (PMC7192512; doi:10.1371/journal.pbio.3000465)
Supplement: S1 Table — (PDF) [file pbio.3000465.s009.pdf]

S1 Table: Abundance of the focal *E. coli* strain in treatments with and without ampicillin after 24h and averaged over the entire experiment. *Treatment* indicates the different experimental groups (Basal is basal medium only; -Comm is sterilized faecal slurry; +Comm is "live" faecal slurry; -Amp and +Amp are with/without ampicillin). *Human donor* gives the human donor that faecal samples used in each treatment came from. CFU_{24h} gives the mean and standard deviation of focal strain abundance after 24h. $Reduction_{24h}$ gives the average reduction in the presence of ampicillin relative to the absence of ampicillin for each combination of human donor and community. $CFU_{expt.}$ and $Reduction_{expt.}$ give the same but averaged across the entire seven-day experiment.

Treatment	Human donor	CFU_{24h} (mean ±s.d.)		$Reduction_{24h}$ (%)	$CFU_{expt.}$ (mean ±s.d.)		$Reduction_{expt.}$ (%)
Basal -Amp	None	4.46×10^7	$\pm6.07\times10^6$		8.94×10^7	$\pm3.85\times10^7$	
Basal +Amp	None	1.38×10^7	$\pm2.68\times10^6$	69.028	3.54×10^7	$\pm2.18\times10^7$	60.350
-Comm -Amp	1	1.33×10^8	$\pm7.21\times10^6$		1.35×10^8	$\pm6.58\times10^7$	
-Comm +Amp	1	1.34×10^7	$\pm2.91\times10^6$	89.955	3.03×10^7	$\pm1.97\times10^7$	77.476
-Comm -Amp	2	1.58×10^8	$\pm3.22\times10^7$		1.43×10^8	$\pm7.30\times10^7$	
-Comm +Amp	2	3.22×10^7	$\pm6.52\times10^6$	79.594	4.75×10^7	$\pm2.21\times10^7$	66.725
-Comm -Amp	3	2.04×10^8	$\pm2.95\times10^7$		1.24×10^8	$\pm6.78\times10^7$	
-Comm +Amp	3	4.43×10^7	$\pm8.59\times10^6$	78.322	3.27×10^7	$\pm1.94\times10^7$	73.743
+Comm -Amp	1	6.41×10^7	$\pm1.08\times10^7$		2.30×10^7	$\pm3.02\times10^7$	
+Comm +Amp	1	8.22×10^3	$\pm1.02\times10^4$	99.987	1.17×10^3	$\pm3.11\times10^3$	99.995
+Comm -Amp	2	9.25×10^7	$\pm4.85\times10^7$		1.09×10^8	$\pm5.86\times10^7$	
+Comm +Amp	2	4.23×10^3	$\pm4.84\times10^3$	99.995	7.30×10^4	$\pm1.57\times10^5$	99.933
+Comm -Amp	3	4.40×10^6	$\pm3.03\times10^6$		5.74×10^7	$\pm5.15\times10^7$	
+Comm +Amp	3	7.08×10^5	$\pm3.60\times10^5$	83.909	1.31×10^7	$\pm3.00\times10^7$	77.226
