# Supplementary material for: Resident microbial communities inhibit growth and antibiotic-resistance evolution of Escherichia coli in human gut microbiome samples
Source: PLoS Biol. 2020 Apr 20;18(4):e3000465. doi: 10.1371/journal.pbio.3000465 (PMC7192512; doi:10.1371/journal.pbio.3000465)
Supplement: S2 Table — qPCR, quantitative PCR. (PDF) [file pbio.3000465.s010.pdf]

S2 Table: Fraction of focal strain on total E.coli abundance determined by qPCR and a mixed calculation based of colony PCR, flow cytometry and amplicon data. *Treatment* and *Human donor* indicate the same experimental groups as in Sup. table 1. *Time point* gives the time after the start of the experiment when the samples were isolated. Frequency of the focal strain compared to total *E. coli* abundance was either determined by qPCR with primers specific for focal strain and *E.coli* or a mixed calculation of abundance of focal strain based on colony PCR, total bacterial abundance measured by flow cytometry and total *E. coli frequency* based on amplicon data. *E.coli* frequency based on total Enterobacteriaceae abundance was determined by reads distribution of the amplicon sequencing results. Frequencies shown are mean values of three replicates and the standard deviation.

			Frequency of focal strain compared to total *E. coli* abundance based on		*E.coli* frequency of total Enterobacteriaceae based on
**Treatment**	**Human donor**	**Time point [h]**	**qPCR (mean % ± s.d.)**	**Colony PCR/ Flow cytometer/ amplicon (mean % ± s.d.)**	**Amplicon (mean % ± s.d.)**
+Comm -Amp	1	24	20.9 ± 3.1	66.9 ± 5.9	99.997 ± 0.003
+Comm +Amp	1	24	0.2 ± 0.1	0.1 ± 0.03	99.93 ± 0.007
+Comm -Amp	2	24	35.1 ± 11.1	27.9 ± 8.3	99.97 ± 0.02
+Comm +Amp	2	24	0.07 ± 0.1	0.004 ± 0.004	99.99 ± 0.006
+Comm -Amp	3	24	18.6 ± 0.6	16.6 ± 3.8	99.999 ± 0.001
+Comm +Amp	3	24	12.9 ± 2.6	8.6 ± 2.5	99.998 ± 0.004
+Comm -Amp	1	168	ND	0	100 ± 0.001
+Comm +Amp	1	168	ND	0	99.999 ± 0.001
+Comm -Amp	2	168	9.6 ± 0.1	32.9 ± 2.5	99.773 ± 0.2
+Comm +Amp	2	168	0.02 ± 0.001	0.0001± 0.0001	99.474 ± 0.64
+Comm -Amp	3	168	9.4± 7.0	23.3± 5.6	99.996 ± 0.007
+Comm +Amp	3	168	ND	0	99.992 ± 0.01
