# Supplementary material for: Resident microbial communities inhibit growth and antibiotic-resistance evolution of Escherichia coli in human gut microbiome samples
Source: PLoS Biol. 2020 Apr 20;18(4):e3000465. doi: 10.1371/journal.pbio.3000465 (PMC7192512; doi:10.1371/journal.pbio.3000465)
Supplement: S3 Table — (PDF) [file pbio.3000465.s011.pdf]

S3 Table (continued on next page): Genomic variants found in randomly selected colony isolates of the focal strain picked from ampicillin-free agar plates at the end of the experiment. *Treatment group* indicates if strain evolved in presence or absence of community (Com) or ampicillin (Amp). *Human donor* gives the origin of the faecal samples. Arrows indicate that intergenic region, list of genes indicate that several genes were affected by the same mutation and delta sign indicates larger deletion on the chromosome.

Treatment group	Human donor	Replicate	*arpA <> iclR*	*casA <> ygcB*	*clsA*	*entD*	*frsA <> phoE*	*gatY <> fbaB*	*gatZ*	*gfvA+B+C, insA+B*	*gtrS*	*hsdS*	*insI1, insX*	*insN*	*intD*	*intD, xisD,exoD, peaD*	*iraM <> ycgX*	*mmuM+P, afuB+C,*	*ompF <> asnS*	*ompR*	*ompT*	*opgB*	*rpoD*	*serS*	*tfaD*	*tfaD <> ybcY*	*tfaX <> appY*	*waaS*	*waaU*	*wbbK*	*wecB*	*yaiO*	*yaiP*	*yaiS <> tauA*	*ybbD*	*ybcy*	*ybfK <> kdpE*	*yddK*	*ydjO*	*yedR <> yedS*	*yfcV <> sixA*	*yfjl*	*ygeF*	*yhhI*	*yibV*	*yjbS*	*yjgL*	*yjhB*	*yjiS <> yjiT*	Δ 566060-584707	Δ 568024-586058
		1												x																																			
Basal -Amp	None	2											▲					▲														x																			
		3	No mutations detected																																																
		1	No mutations detected																																																
-Com -Amp	1	2																						●																											
		3	No mutations detected																																																
		1																																															
Com -Amp	1	2	Focal strain below detection limit																																																
		3																																															
		1	No mutations detected																																																
-Com -Amp	2	2																																															
		3									▲											●									●																				
		1																																															
+Com -Amp	2	2	No mutations detected																																																
		3																																															
		1																																						x									
-Com -Amp	3	2									▲																		▲					▲										x							
		3	No mutations detected																																																
		1								x					●						●				●	●										●														▲	
+Com -Amp	3	2												x																																			
		3				x	●									▲											▲						x					●													▲

▲ Deletion x Insertion ● SNP

Continuation S3 Table: Genomic variants found in randomly selected colony isolates of the focal strain picked from ampicillin-free agar plates at the end of the experiment.

Treatment group	Human donor	Replicate	arpA <> iclR	casA <> ygcB	clsA	entD	frsA <> phoE	gatY <> fbaB	gatZ	gfvA+B+C, insA+B	gtrS	hsdS	insI1, insX	insN	intD	intD, xisD,exoD,	iraM <> ycgX	mmuM+P, afuB+C,	ompF <> asnS	ompR	ompT	opgB	rpoD	serS	tfaD	tfaD <> ybcY	tfaX <> appY	waaS	waaU	wbbK	wecB	yaiO	yaiP	yaiS <> tauA	ybbD	ybcy	ybfK <> kdpE	yddK	ydjO	yedR <> yedS	yfcV <> sixA	yfjl	ygeF	yhhI	yibV	yjbS	yjgL	yjhB	yjiS <> yjiT	Δ 566060-584707	Δ 568024-586058
Basal +Amp	None	1												x																																					
		2	No mutations detected																																																
		3																																																	
-Com +Amp	1	1																					●												▲					▲		x									
		2	No mutations detected																																																
		3												x						●																															
+Com +Amp	1	1	Focal strain below detection limit																																																
		2																																																	
		3																																																	
-Com +Amp	2	1									▲								x																																
		2	▲		x															●																								▲			▲			
		3						●												●							▲																								
+Com +Amp	2	1																																																	
		2		▲													▲											▲	▲	▲							▲		▲		▲		▲			▲	▲				
		3									▲																																						▲		
-Com +Amp	3	1												x																																	▲			
		2	No mutations detected																																																
		3																																																	
+Com +Amp	3	1	Focal strain below detection limit																																																
		2																																																	
		3																																																	

▲ Deletion   x Insertion   ● SNP
