# Supplementary material for: Resident microbial communities inhibit growth and antibiotic-resistance evolution of Escherichia coli in human gut microbiome samples
Source: PLoS Biol. 2020 Apr 20;18(4):e3000465. doi: 10.1371/journal.pbio.3000465 (PMC7192512; doi:10.1371/journal.pbio.3000465)
Supplement: S4 Table — (PDF) [file pbio.3000465.s012.pdf]

S4 Table: Antibiotic resistance genes, plasmid replicons and genes involved in conjugative transfer and formation of type VI secretion system found on plasmid 1 of isolates from human donor 1 resident *E. coli* community and on the chromosome of human donor 3 resident *E. coli* isolates of each replicate population.

		Plasmid 1 from human donor 1 without antibiotics of replicate community:			Plasmid 1 from human donor 1 with antibiotics of replicate community:			Chromosome from human donor 3 without antibiotics of replicate community:			Chromosome from human donor 3 with antibiotics of replicate community:		
**Genomic feature**	**Function**	1	2	3	1	2	3	1	2	3	1	2	3
blaTem-1b	Beta lactam resistance	✓	✓	✓	✓	✓	✓	✓	✓	✓	✓	✓	✓
aph(3)	Aminoglycoside resistance	✓	✓	✓	✓	✓	✓	✓	✓	✓	✓	✓	✓
aph(6)	Aminoglycoside resistance	✓	✓	✓	✓	✓	✓	✓	✓	✓	✓	✓	✓
sul2	Sulphonamide resistance	✓	✓	✓	✓	✓	✓	✓	✓	✓	✓	✓	✓
tet(A)	Tetracycline resistance	✕	✕	✕	✕	✕	✕	✓	✓	✓	✓	✓	✓
IncQ1	Replicon	✕	✕	✕	✕	✕	✕	✓	✓	✓	✓	✓	✓
IncFIC(FII)	Replicon	✓	✓	✓	✓	✓	✓	✕	✕	✕	✕	✕	✕
IncFIA	Replicon	✓	✓	✓	✓	✓	✓	✕	✕	✕	✕	✕	✕
IncFIB	Replicon	✓	✓	✓	✓	✓	✓	✕	✕	✕	✕	✕	✕
tra genes [a]	Involved in conjugative transfer	✓	✓	✓	✓	✓	✓	✕	✕	✕	✕	✕	✕
tss genes [a]	Type 6 secretion system	✓	✓	✓	✓	✓	✓	✓	✓	✓	✓	✓	✓

[a] For names of involved genes see Fig. 4
