# Supplementary material for: Resident microbial communities inhibit growth and antibiotic-resistance evolution of Escherichia coli in human gut microbiome samples
Source: PLoS Biol. 2020 Apr 20;18(4):e3000465. doi: 10.1371/journal.pbio.3000465 (PMC7192512; doi:10.1371/journal.pbio.3000465)
Supplement: S5 Table — IC90, concentration required to reduce growth by 90%. (PDF) [file pbio.3000465.s013.pdf]

S5 Table: IC90 values of ancestor and ampicillin resistant evolved strains. IC90 was defined as the concentration where the OD value was less than 10% of the untreated control.

Isolate	Human donor	Isolated from Replicate population	IC90 concentration [µg/ml]
Ancestor focal strain	None	None	8
Basal +Amp	None	1	20
Basal +Amp	None	2	26
Basal +Amp	None	3	16
-Com +Amp	1	1	20
-Com +Amp	1	2	16
-Com +Amp	1	3	16
-Com +Amp	3	1	20
-Com +Amp	3	2	20
Ancestor transconjugant	None	None	8
Transconjugant	None	None	>60
Resident E. coli	1	None	>60
