# Supplementary material for: Resident microbial communities inhibit growth and antibiotic-resistance evolution of Escherichia coli in human gut microbiome samples
Source: PLoS Biol. 2020 Apr 20;18(4):e3000465. doi: 10.1371/journal.pbio.3000465 (PMC7192512; doi:10.1371/journal.pbio.3000465)
Supplement: S6 Table — (PDF) [file pbio.3000465.s014.pdf]

S6 Table: List of all sequenced isolates: *Treatment, Human donor* and *Replicate population* indicates where the strain was isolated from. Strains were isolated either from ampicillin-free or ampicillin containing plates. *Strain* indicates if it is the focal or resident strain. *Sample ID* gives the short name used for sequencing and *Platform* indicates if the isolate was sequenced with Illumnia or MinION. *ENA accession* gives the accession number of the isolate in the repository and *Ampicillin resistant* indicates if the isolate could grow at the MIC of the ancestor strain.

Treatment	Human donor	Replicate population	Isolated from	Strain	Sample ID	Platform	ENA Accession	Ampicillin resistant
Basal -Amp	none	1	Ampicillin-free plates	Focal	Strep_B1	Illumina	ERS4256211	No
Basal -Amp	none	2	Ampicillin-free plates	Focal	Strep_B2	Illumina	ERS4256212	No
Basal -Amp	none	3	Ampicillin-free plates	Focal	Strep_B3	Illumina	ERS4256213	No
Basal +Amp	none	1	Ampicillin-free plates	Focal	Strep_BA1	Illumina	ERS4256214	No
Basal +Amp	none	2	Ampicillin-free plates	Focal	Strep_BA2	Illumina	ERS4256215	No
Basal +Amp	none	3	Ampicillin-free plates	Focal	Strep_BA3	Illumina	ERS4256216	No
-Com -Amp	1	1	Ampicillin-free plates	Focal	Strep_D1S1	Illumina	ERS4256217	No
-Com -Amp	1	2	Ampicillin-free plates	Focal	Strep_D1S2	Illumina	ERS4256218	No
-Com -Amp	1	3	Ampicillin-free plates	Focal	Strep_D1S3	Illumina	ERS4256219	No
-Com +Amp	1	1	Ampicillin-free plates	Focal	Strep_D1SA1	Illumina	ERS4256220	Yes
-Com +Amp	1	2	Ampicillin-free plates	Focal	Strep_D1SA2	Illumina	ERS4256221	No
-Com +Amp	1	3	Ampicillin-free plates	Focal	Strep_D1SA3	Illumina	ERS4256222	No
-Com -Amp	2	1	Ampicillin-free plates	Focal	Strep_D2S1	Illumina	ERS4256229	No
-Com -Amp	2	2	Ampicillin-free plates	Focal	Strep_D2S2	Illumina	ERS4256230	No
-Com -Amp	2	3	Ampicillin-free plates	Focal	Strep_D2S3	Illumina	ERS4256231	No
-Com +Amp	2	1	Ampicillin-free plates	Focal	Strep_D2SA1	Illumina	ERS4256232	No
-Com +Amp	2	2	Ampicillin-free plates	Focal	Strep_D2SA2	Illumina	ERS4256233	No
-Com +Amp	2	3	Ampicillin-free plates	Focal	Strep_D2SA3	Illumina	ERS4256234	No
+Com -Amp	2	1	Ampicillin-free plates	Focal	Strep_D2C1	Illumina	ERS4256223	No
+Com -Amp	2	2	Ampicillin-free plates	Focal	Strep_D2C2	Illumina	ERS4256224	No
+Com -Amp	2	3	Ampicillin-free plates	Focal	Strep_D2C3	Illumina	ERS4256225	No
+Com +Amp	2	1	Ampicillin-free plates	Focal	Strep_D2CA1	Illumina	ERS4256226	No
+Com +Amp	2	2	Ampicillin-free plates	Focal	Strep_D2CA2	Illumina	ERS4256227	No

+Com +Amp	2	3	Ampicillin-free plates	Focal	Strep_D2CA3	Illumina	ERS4256228	No
-Com -Amp	3	1	Ampicillin-free plates	Focal	Strep_D3S1	Illumina	ERS4256238	No
-Com -Amp	3	2	Ampicillin-free plates	Focal	Strep_D3S2	Illumina	ERS4256239	No
-Com -Amp	3	3	Ampicillin-free plates	Focal	Strep_D3S3	Illumina	ERS4256240	No
-Com +Amp	3	1	Ampicillin-free plates	Focal	Strep_D3SA1	Illumina	ERS4256241	No
-Com +Amp	3	2	Ampicillin-free plates	Focal	Strep_D3SA2	Illumina	ERS4256242	No
-Com +Amp	3	3	Ampicillin-free plates	Focal	Strep_D3SA3	Illumina	ERS4256243	No
+Com -Amp	3	1	Ampicillin-free plates	Focal	Strep_D3C1	Illumina	ERS4256235	No
+Com -Amp	3	2	Ampicillin-free plates	Focal	Strep_D3C2	Illumina	ERS4256236	No
+Com -Amp	3	3	Ampicillin-free plates	Focal	Strep_D3C3	Illumina	ERS4256237	No
Basal +Amp	none	1	Ampicillin plates	Focal	Amp_BA1	Illumina	ERS4256244	Yes
Basal +Amp	none	2	Ampicillin plates	Focal	Amp_BA2	Illumina	ERS4256245	Yes
Basal +Amp	none	3	Ampicillin plates	Focal	Amp_BA3	Illumina	ERS4256246	Yes
+Com +Amp	1	1	Ampicillin plates	Focal	Amp_D1SA1	Illumina	ERS4256247	Yes
+Com +Amp	1	2	Ampicillin plates	Focal	Amp_D1SA2	Illumina	ERS4256248	Yes
+Com +Amp	1	3	Ampicillin plates	Focal	Amp_D1SA3	Illumina	ERS4256249	Yes
+Com +Amp	3	1	Ampicillin plates	Focal	Amp_D3SA1	Illumina	ERS4256250	Yes
+Com +Amp	3	2	Ampicillin plates	Focal	Amp_D3SA2	Illumina	ERS4256251	Yes
+Com -Amp	1	1	Ampicillin plates	Resident	Resident Ecoli D1C1	Illumina	ERS4256252	Yes
+Com -Amp	1	2	Ampicillin plates	Resident	Resident Ecoli D1C2	Illumina	ERS4256253	Yes
+Com -Amp	1	3	Ampicillin plates	Resident	Resident Ecoli D1C3	Illumina	ERS4256254	Yes
+Com +Amp	1	1	Ampicillin plates	Resident	Resident Ecoli D1CA1	Illumina	ERS4256255	Yes
+Com +Amp	1	2	Ampicillin plates	Resident	Resident Ecoli D1CA2	Illumina	ERS4256256	Yes
+Com +Amp	1	3	Ampicillin plates	Resident	Resident Ecoli D1CA3	Illumina	ERS4256257	Yes
+Com -Amp	3	1	Ampicillin plates	Resident	Resident Ecoli D3C1	Illumina	ERS4256258	Yes
+Com -Amp	3	2	Ampicillin plates	Resident	Resident Ecoli D3C2	Illumina	ERS4256259	Yes
+Com -Amp	3	3	Ampicillin plates	Resident	Resident Ecoli D3C3	Illumina	ERS4256260	Yes
+Com +Amp	3	1	Ampicillin plates	Resident	Resident Ecoli D3CA1	Illumina	ERS4256261	Yes
+Com +Amp	3	2	Ampicillin plates	Resident	Resident Ecoli D3CA2	Illumina	ERS4256262	Yes
+Com +Amp	3	3	Ampicillin plates	Resident	Resident Ecoli D3CA3	Illumina	ERS4256263	Yes

+Com +Amp	1	1	Ampicillin plates	Resident	Resident Ecoli D1CA1	MinION	ERS4256375	Yes
+Com +Amp	3	1	Ampicillin plates	Resident	Resident Ecoli D3CA1	MinION	ERS4256376	Yes
