# Supplementary material for: Resident microbial communities inhibit growth and antibiotic-resistance evolution of Escherichia coli in human gut microbiome samples
Source: PLoS Biol. 2020 Apr 20;18(4):e3000465. doi: 10.1371/journal.pbio.3000465 (PMC7192512; doi:10.1371/journal.pbio.3000465)
Supplement: S7 Table — (PDF) [file pbio.3000465.s015.pdf]

S7 Table A: Assembly statistics for genome sequencing on Illumina platform of resident *E. coli* isolated from the resident microbiota of human donors 1 and 3. *Ampicillin treatment*, *Human donor* and *Replicate microcosm* indicate the treatment group of the main experiment each isolate was taken from before sequencing.

Ampicillin treatment	Human donor	Replicate microcosm	No. contigs	Total genome length [bp]	Largest contig [bp]	N50	L50	GC (%)
-Amp	1	1	88	5214174	557565	207210	8	50.5
-Amp	1	2	103	5185519	334964	153591	12	50.5
-Amp	1	3	115	5207591	346088	158712	12	50.5
+Amp	1	1	4	5330642	5158979	5158979	1	50.5
+Amp	1	1	86	5218659	522040	196713	8	50.5
+Amp	1	2	99	5207712	522040	162556	10	50.5
+Amp	1	3	91	5216794	520971	189113	9	50.5
-Amp	3	1	95	5095638	570677	191144	9	50.6
-Amp	3	2	110	5094217	570677	191168	9	50.6
-Amp	3	3	86	5096162	540821	218238	8	50.6
+Amp	3	1	4	5186270	5072055	5072055	1	50.6
+Amp	3	1	105	5100479	373745	203252	10	50.6
+Amp	3	2	93	5093766	570638	191168	9	50.6
+Amp	3	3	97	5099961	570542	203252	9	50.6

S7 Table B: Assembly statistics for genome sequencing on MinION platform of resident *E. coli* isolated from the resident microbiota of human donors 1 and 3. *Ampicillin treatment*, *Human donor* and *Replicate microcosm* indicate the treatment group of the main experiment each isolate was taken from before sequencing and *Circular* indicates if the contig was closed or not.

Ampicillin treatment	Human donor	Replicate microcosm	Contig 1		Contig 2		Contig 3		Contig 4		Contig 5	
			Length [bp]	Circular	Length [bp]	Circular	Length [bp]	Circular	Length [bp]	Circular	Length [bp]	Circular
+Amp	1	1	5158979	Yes	163562	Yes	6647	Yes	1454	No	-	-
+Amp	3	1	5072055	No	109229	No	3030	No	1584	No	372	No
