# Supplementary material for: Resident microbial communities inhibit growth and antibiotic-resistance evolution of Escherichia coli in human gut microbiome samples
Source: PLoS Biol. 2020 Apr 20;18(4):e3000465. doi: 10.1371/journal.pbio.3000465 (PMC7192512; doi:10.1371/journal.pbio.3000465)
Supplement: Attachment — Submitted filename: Response_letter_blactam_gut_microcosm.docx [file pbio.3000465.s018.docx]

Dear Dr. Richardson, Academic Editor and Reviewers,

Thank you for the helpful feedback, which has helped us improve the manuscript. We have addressed all comments, including additional experiments, analyses and simulations. We provide a summary of our revisions below after the editor's suggestions. This is followed by a point-by-point response to the reviewers' comments (editor and reviewer comments are shown in blue italics).

Sincerely,

Michael Baumgartner (on behalf of all authors)

*Of particular note, the Academic Editor highlights the need to: 1) determine the role of inoculum size and/or antibiotic concentration in the outcome of resistance evolution, 2) contextualize the results of the study with the well-known concept of colonization resistance and better highlight what is known and what is new, 3) provide a better quantification of population abundances, 4) clarify, or at least further discuss, the importance of specific microbiota compositions to the outcome of resistance evolution. Due to the small sample size of fecal microbiota samples used, it is difficult to make strong general conclusions and the limitations must be made clear, as noted by Rev #3. While the Academic Editor appreciates that addressing the point raised by Rev #2 in point #2 (request for assaying temporal stability of bacteria and to explain the differences between donors) would improve the manuscript, we will not require this data in a revision.*

We have now carried out additional experiments and revised the text as suggested. In summary we:

I) Resolved whether there is an 'inoculum effect' for ampicillin at high cell densities in our experimental system, and put this in the context of how ampicillin affected population densities and selection for resistance (S8 Fig; Line 450-454).
II) Revised our interpretation and presentation of the results to account for variation among human donors more explicitly (lines 117-120; 149-164; 223-232), and put our findings in the context of colonization resistance (Line 400-402 and 419-442).
III) Did competition assays in the presence/absence of resident microbiota and at sub-/supra-MIC antibiotic concentrations (S5 Fig; Line 337-368). This allowed us to quantify costs and benefits of resistance mutations and plasmids from the main experiment. We found resident microbiota amplified the costs of certain resistance mutations, but amplified the benefits of plasmids we identified in resident *E. coli* strains. This helps to explain the main findings we reported previously.
IV) Used qPCR to gain a more granular understanding of the frequency of our focal strain compared to other *E. coli*, and to quantify the abundance of the conjugative plasmid in the human donor 1 background (S3 Fig; S2 Table; Line 223-232).
V) Used a mathematical model to simulate the population dynamics, improving our understanding of how resident microbiota can affect the spread of conjugatively transferable plasmids (S1 Model; Line 383-391).
VI) Performed long-read sequencing with the MinION platform for the resident *E. coli* isolates found in the samples from human donors 1 and 3 (Fig 4; Line 287-303). This revealed the isolate of human donor 1 contained two plasmids, one with antibiotic resistance genes, whereas in the isolate from human donor 3 the resistance genes were integrated on the chromosome.

*Reviewer #1: 

Baumgartner et al present new findings regarding the evolution and acquisition of antibiotic resistance as it relates to the interplay between a resident microbial community and an invading strain. The authors used an ex vivo fecal slurry culture system that allows a degree of parallelism and replicability that is hard to recapitulate with animal models. They find that the focal strain of E. coli evolves resistance to ampicillin in the absence of a microbial community but not in its presence, and that failure to mobilize plasmids bearing antibiotic resistance from endogenous E. coli strains is likely one underlying cause. 

Generally, I am interested by and like the study, and I believe that the development of ex vivo models is important to advance the mechanistic understanding of microbiome biology. The statistical analyses appear sound. However, I find parts of the study unsatisfying.

Major comments:

1. It is not very surprising to me that the evolution of antibiotic resistance depends on selection for mutations arising within a population, and that limitation of population size restricts the availability of mutants for selection to act on. This appears to be what is occurring for Donors 2 and 3 in the presence of antibiotics. The authors state “Given the variety and likely common occurrence of mechanisms that can generate such suppression of invaders (e.g. resource competition), these types of effects are likely common in species-rich communities such as the mammalian gastrointestinal tract.” If the observed phenotype in donors 2 and 3 + amp was due to resource competition, why was it not observed in the no antibiotic condition?

I can see at least two possibilities for what could be occurring for these samples. First, exposure of the focal strain to the community results in sensitization to antibiotics. Second, the focal strain, while normally able to resist inhibition by the community, is destabilized by antibiotics such that colonization resistance by the community is unmasked.  

The first possibility could potentially be addressed by taking isolates from each timepoint from these samples and performing assays such as those presented in Fig 5A, the hypothesis being that exposure to the community results in increased sensitization to antibiotics over time. The second point could potentially be addressed by asking if a pulse of antibiotics then removal results in a similar subsequent elimination of the focal strain, or if persistent addition of antibiotics is required.*

In our main experiment, suppression of the focal strain by ampicillin was stronger in the community treatments. In the revision we have better explained this effect, including how it varies among human donors (S1 Table; Line 117-125 in the main text). We also include new competition experiments to measure how the community changes the relative fitness of individual strains (including focal strain isolates from the main experiment and transconjugants we made using plasmids isolated from the community), in both the presence and absence of ampicillin (S5+S6 Fig; Line 337-368). This confirmed the community changes the strength of antibiotic inhibition, and revealed this makes resistance mutations we observed in the community-free treatments less beneficial, but plasmids more beneficial. Our MIC data (Fig 5A) suggest this probably reflects the relatively weak resistance conferred by these mutations, rendering them beneficial only in the absence of the community. This helps to explain our findings for resistance evolution in the main experiment.

In further new experiments we used competition assays to show that resident *E. coli* strains from human donors 1 and 3 had high competitive fitness, as well as high antibiotic resistance (Fig 5A and S7 Fig; Line 368-370). This, as well as our more in-depth analysis of changes in the resident microbiota during the main experiment (Line 200-204; S2 Table), helps to better understand why our focal strain was outcompeted or excluded in the main experiment. This is further supported by our revised genomic analysis of resident *E. coli* (Fig 4, S4 Table).

We hope these revisions make the main results about how resident microbiota modify selection on antibiotic resistance clearer, and what this means for the spread of particular mutations and plasmids.

*2.	The plasmid from Donor 1 was able to be transferred in vitro on agar but not in liquid culture or in the static incubator. Since conjugation and mobilization of plasmids does indeed occur in vivo (for example, in Stecher et al cited in ref. 55), I am concerned that this lack of mobilization merely reflects the unsuitability of the static incubation culture system for modeling the contact-dependent interactions required for conjugation that occur in the physiological context of the gut. To this point, static incubation in a Hungate tube may not approximate the gut environment, where presumably mixing through effects of peristalsis or motility take place, and some bacteria could inhabit biofilms where horizontal gene transfer is common (De Vos 2015, NPJ Biofilms and Microbiomes; Stalder and Top 2016, NPJ Biofilms and Microbiomes).*

We agree the physical constraints on plasmid transfer in our experiment (surface vs liquid) will not affect all plasmids or in all environments. Our finding that this prevented focal *E. coli* from acquiring beneficial plasmids, leading to colonization resistance rather than antibiotic resistance evolution, nevertheless has important implications. First, it is known that some other plasmids are subject to similar constraints on transfer (discussed on Lines 470-487), and we can expect bacteria in nature to encounter environments that are sometimes relatively well mixed and other times relatively spatially structured. Therefore the physical barriers that affected transfer here are likely not exclusive to our system. Second, the more general point that transfer dynamics depend not only on donor and recipient abundances and the maximum transfer rate, but also on other environmental factors, suggests predicting the spread of plasmids in vivo requires more than genomic or metagenomic information about which resistance genes are present on which plasmids, it also requires information about the sensitivity of conjugation to local abiotic conditions.

*Another point relevant here is that only in Donor 1 did the focal strain decrease in abundance in the absence of antibiotics suggesting that the endogenous microbiota were particularly “good” at suppressing the growth of the focal strain.*

We have added more explanation and revised our analyses to describe the differences depending on human donor more clearly (Lines 200-231). Our revised genomic analysis (Fig 4; Lines 287-303) and competition experiments with resident *E. coli* from human donor 1 (Lines 368-370; S7 Fig) provide additional information that helps to explain why the focal strain was so rapidly outcompeted in these microcosms (e.g. type 6 secretion systems and strong competitive advantage of resident *E. coli* shown in Fig 4 and S7 Fig).

*Decreased growth corresponds to fewer cells to partake in conjugative transfer (and hence perhaps the failure to recover resistant colonies), yet the data in Fig 5B suggests that the culture conditions are the primary effect.*

We now include competition experiments showing directly that the plasmid would have been highly beneficial for the focal strain in these conditions (S5+S6 Fig, lines 337-368), indicating it was indeed the lack of transfer rather the lack of a benefit that prevented it from spreading in the main experiment. We have also added simulations showing that even if the plasmid had been transferable in our experimental system, suppression of the focal strain by the resident community would have strongly affected the likelihood of transconjugants reaching appreciable numbers (S1 Model, lines 383-391).

*3.	I think it would be very useful to have a more granular understanding of the Enterobacteriaceae expansion at the species level (or even just at the level of total E. coli abundance). The relative abundance taxonomic data show an increase in Enterobacteriaceae in the later timepoint, to between ~25-50%. The authors state that this includes the focal E. coli strain, but at between 0-30% of the total Enterobacteriaceae. What proportion of the Enterobacteriaceae are endogenous E. coli? Is expansion of endogenous E. coli driving the overall increase across donors? This could be accomplished by qPCR analysis, for example.*

We have taken three steps to clarify this. First, we looked in more detail at the reads distribution of the amplicon data, providing information about the species distribution within the *Enterobacteriaceae*. This showed *E. coli* was the main contributor to this family (Lines 200-204 and S2 Table). Second, to gain a better understanding of how the focal strain changed over time compared to total *E. coli*, we performed qPCR as suggested (Lines 223-232; S3 Fig). Third, we provide more information about focal strain abundance relative to other *E. coli* inferred from plating, flow cytometry and amplicon data. This was referred to in the previous version, but not in sufficient detail. These data show a similar pattern to the qPCR data (228-231; S2 Table).

*Minor comments: 

1.	The amount of detail in the figure legends could be improved. An example is Fig 5A, where the assay itself is not described. Extrapolating from the methods, the authors took 1ul of an overnight culture and diluted it 1:100 in media containing antibiotic. Is the starting OD600 really ~0.75 after a 1:100 dilution from an overnight? A more traditional way to do this experiment would be to back dilute further, allow cultures to grow to a defined OD600 value (say 0.6), before adding antibiotic. This ensures that viable cell counts from exponential phase are more consistent across conditions.*

We have revised this and the other figure legends to provide more detail. In this case (Fig 5A), it is the final OD600 that is shown (a measure of bacterial density after 24h of growth).

*2.	The plot in Figure 3 is difficult to look at (axis labels are challenging to read due to density of text). I suggest limiting the text somehow, or moving this to supplemental material.*

We re-organized Figure 3, including less dense labels on the axes, to make it easier to read. *3.	The authors do not cite any of the numerous studies utilizing metagenomics to assess the mobility of antibiotic resistance genes in humans. For instance, Jiang, Alm and colleagues used metagenomic analyses to report that antibiotic resistance often spreads through mobile genetic elements in human gut samples (bioRxiv,2017* [*https://doi.org/10.1101/214213*](https://doi.org/10.1101/214213)*).*

We added two additional citations that specifically look at metagenomic data, including the suggested paper by Jiang, Alm and colleagues (Line 78).

--------------


*Reviewer #2: 

The paper by Baumgartner et al. shows how the presence of a resident bacterial community can modulate the suppressive effects of an antibiotic on growth and colonization by an invader, in this case an E. coli focal strain in an experimental in-vitro system. They also compare antibiotic resistance evolution in the focal strain, in the presence and absence of the resident community, and find that in the presence of competition with a resident community, antibiotic resistance evolution was also suppressed. In fact resistance only evolved by chromosomal mutation and in the absence of a resident microbial community. These genetic mutations are described in detail. The lack of resistance evolution by plasmid-transfer in the presence of a resident community could not be explained via lack of available resistance plasmids among such bacteria, and the authors claim in the paper that it may be due to genetic constraints in one case (resident bacteria from Donor 3), and physical
constraints in the other (resident bacteria from Donor 1). The paper is well-written and the experiments and statistical analyses reasonably performed, although some of the results in my view are not extremely novel or surprising, and moreover some of the empirical patterns reported are simply reported and not integrated with each other, leaving unclear what is it that one exactly learns from them. My points are listed below:

1. The main results on colonization resistance from resident microbiota are known for a number of years now, and in in-vivo settings, so the first results obtained by the authors are only incremental, testing colonization resistance in a particular case in vitro.*

We have revised the text in this section to provide more details on the effect of the resident community. One aspect that goes beyond the basic concept of colonization resistance is that the resident community not only suppressed growth of the focal strain, but amplified the effects of antibiotic treatment (now explained in more detail on Lines 117-125 and 445-464; S1 Table). We have added new competition assay data showing this in turn changed the fitness costs and benefits of individual alleles involved in antibiotic resistance (making resistance mutations from the community-free treatments more costly, and plasmids from resident microbiota more beneficial; Lines 337-368; S5 Fig). We hope these new details draw out the novelty more.

*I have a remark about the statistical analyses performed to prove this point in the paper, that all the experiments are analyzed simultaneously in the model (page 6). It is only in this way by aggregating antibiotic-free and antibiotic-treatment dynamics that colonization resistance emerges from the results of this paper. In fact, if one looks at figure 1, it is only in 1/3 of "hosts" or cases, that colonization resistance is observed in the 'baseline regime' without antibiotics. I think unifying all experiments loses the patterns in the data: a more accurate representation and modeling of this data, would be separating the antibiotic-free and antibiotic-treatment results. What is interesting, is that even when
colonization resistance was not observed in the absence of antibiotics, and instead invasion and coexistence occurred, suppressive effects by residents were observed in the presence of antibiotics. Thus antibiotics modulate the effective competition between resident community and invader in donors 2 and 3, and amplify the competitive exclusion in donor 1. I think the author's general mechanical application of statistical modeling to all data in a single shot misses these intricate patterns.*

We have revised our analysis according to this suggestion, breaking it down more by human donor and antibiotic treatment (Lines 138-167). This highlights the variation among human donors, though we also note there are some key patterns that apply across all three human donors (e.g. suppression of focal strain growth, which is strongest in the presence of ampicillin; discussed on Lines 445-455). Because our experimental design was fully factorial, we first analyze the entire dataset, and only after showing there are significant interactions between the factors do we dive into analyzing subsets of the data (different donors and/or antibiotic treatments; Lines 117-167), in line with conventional practice for this type of linear model. *2. The other results about total bacterial abundance and diversity. 
- Did the authors perform any test on the stability of total bacterial abundance in the absence of antibiotics?*

The closest we have is flow cytometry data on total bacterial abundance over time, including in the absence of antibiotics (Lines 169-182; Fig 2). In the revised manuscript we now also provide more detail on how the focal strain and other *E. coli* contributed to these changes over time (Lines 200-204; 223-232; S2 Table; S3 Fig).

*- The fact that antibiotic effect was time-dependent is nothing new, it is expected and has been shown from mechanistic models of bacteria-antibiotic interaction and also experimentally (see e.g. the Regoes paper on the pharmacodynamic model 2001) so applying statistical analyses to this phenomenon brings nothing really new, only shows that such an expected pattern applies also here.*

We leave this in because the antibiotic effect is important for understanding selection on resistance. However the revised manuscript puts more emphasis on two novel aspects: the altered strength of antibiotic inhibition for the focal strain in the presence of resident microbiota (Lines 117-125; S1 Table), and how this was linked to altered fitness costs and benefits of individual resistance mutations and plasmids (Lines 337-368; S5 Fig).

*- How are the differences between Donors  related? So far the paper does not make any attempt to integrate the donor variation across different metrics. Results are presented, differences are shown that exist, but no linkage between them, e.g. via some population dynamic modeling. Or are they unrelated? Just descriptive statistics of the resident community in different cases? Then the question is what did we learn?*

With only three donors, we have limited power to correlate variation depending on human donor with host factors or presence/absence of particular taxa in the corresponding amplicon datasets. We have nevertheless provided a more detailed analysis of the variation among donors in the main experiment (Lines 200-232), including re-organization of Fig 3B (16S data) to make it easier to compare bacterial diversity across the three donors. We also now provide more detailed information about changing abundances of the focal strain relative to resident *E. coli* (S2 Table and S3 Fig), as well as competition data and genomic analyses that help to explain why resident *E. coli* had such an advantage over the focal strain in our main experiment with samples from human donor 1 (Fig 4 and S7 Fig). More importantly, that some key trends were observed across all three human donors, such as stronger suppression of the focal strain by the resident community in the presence of ampicillin, indicates they are repeatable across individual microbiomes.

*- A similar point applies to the changes in diversity over time: time in this dataset emerges to be a stronger predictor of changes in diversity than presence/absence of antibiotics. What is the meaning of this? The authors just report this result, but do not explain or interpret its biological consequence. This finding suggests the selection going on in the absence of antibiotics (to in-vitro experimental conditions) is stronger than the selective pressure exerted by the antibiotics. Does this have to do with the dose used, which was sub-MIC? This could be important given other literature findings that antibiotics reduce drastically microbiota diversity. Links with clinical/epidemiological findings actually are important, and should be made here.*

We expanded our discussion of this (lines 489-504). We also added new data on how the fitness of the focal strain changes if we increase the concentration to higher than the MIC (S5 Fig; lines 337-368). This is consistent with the idea that shifts in community composition resulting from antibiotic application would probably be more severe at higher antibiotic concentrations (discussed on lines 496-500).

*3. The finding about antibiotic resistance evolution.
I think this is the most interesting part of the paper, and it's here that this article's strength lies: in the what/how/ and why of such patterns. Unfortunately however, I found this section somewhat incomplete. I agree that mechanisms should be studied, as the authors do, but what about population dynamics of resistance transfer? Efficient evolution via chromosomal mutation or horizontal gene transfer/plasmids depends on the population abundances and frequencies of the donor/recipient cells and corresponding process rates. The authors do not attempt to do any quantification of population dynamics here, and only focus on the molecular constraint and physical constraint, and show that indeed despite the huge beneficial effect of extant plasmids, the transfer did not happen.*

We hope the what/how/why are clearer with the new data we added about costs/benefits of individual resistance mutations and plasmids (S5+S6 Fig, lines 337-368). This shows directly that the plasmid would have been beneficial to the focal strain in the presence of the community and antibiotics, and therefore the lack of transconjugants in the main experiment is better explained by lack of transfer than a lack of fitness benefits. The new data on costs of resistance mutations from the community-free treatments also help to explain why these mutations did not spread in the community treatments. Finally, we added simulations of the population dynamics (S1 Model, lines 383-391). This allowed us to estimate the transconjugant frequencies we would have expected if the plasmid had been transferable in our system, accounting for observed donor/recipient abundances and using a wide range of transfer rates we extracted from the literature. *It would have been useful to have conducted the experiment at one sub-MIC dose as the authors have done, and at one supra-MIC dose, to see whether the selection pressure imposed actually changes the rate of plasmid spread. In retrospect, such resistance, even if it provided huge benefits, did not evolve because (perhaps?) it was not needed at such low selection pressure. A mathematical model would help to quantify some of these expectations.*  

Both our main experiment and follow-up competition experiments indicated ampicillin has an even stronger effect on the focal strain in the presence of the community (Fig 1; S5 Fig and S1 Table). This suggests selection for resistance is in fact stronger in the community treatments, which we explain in more detail in the revised Results (lines 117-120; S1 Table). More importantly, in light of this suggestion we included a supra-MIC treatment in our new competition assays (S5 Fig, lines 337-368). This showed increased ampicillin concentration had a strong detrimental effect on focal strain survival and, as at the lower concentration we used in the main experiment, uptake of the plasmid would have provided a large fitness benefit to the focal strain. We accounted for the observed fitness effects in our simulations of the population dynamics and transfer of hypothetical conjugative plasmids (S1 Model, lines 383-391).

*I also find the listing of genetic mutations (in parallel or specific) that occurred in the absence of antibiotics something that did not add much to the main point of this paper, which in my view is about interaction between resident and invader. Now why did no such chromosomal mutations evolve in the mixed cases? Is it because they have a very strong fitness cost in competition? Can this be tested? How? As these results stand now, they do not add much insights to the main point of this paper.* 

We have now tested whether focal strain isolates that evolved in the community-free treatment had a fitness benefit relative to the ancestral strain in the presence/absence of the community and at various ampicillin concentrations (S5 Fig, lines 337-368). These data suggest resistance mutations detected in community-free treatments were not beneficial in the community treatments, which can be explained by the relatively small increase in resistance they conferred (Fig 5A). This helps to explain why some mutations were restricted to particular treatments, which we mention briefly in the main text (Lines 454-458). Nevertheless, we agree that detailed listing of mutations is potentially distracting from the main story, and have therefore kept most of the details in the Supplementary Information.

*5. Overall I find the paper interesting, showing four very important and unexpected outcomes, that deserve to be more central to the paper:
- no colonization resistance in the absence of antibiotics (figure 1, donors 2,3) suggesting that multiple scenarios are possible depending on donor-invader combinations - but this is not commented upon*

*- plasmid-mediated resistance is not always going to evolve in the invader, even if it is present in a majority background population*

*- competition resident community-invader amplifies the suppressive antibiotic effects on an invader.
-competition resident community-invader reduces the evolutionary potential of invader populations.*

*All the other analyses in my view, are secondary and thus require less focus in the main text.*

In light of these comments we have emphasized these aspects more strongly (revised results section: Lines 147-167 and S1 Table, ; competition data: S5 Fig; simulations: S1 Model).

--------------

*Reviewer #3:* 

*Baumgartner et al present an interesting study on how microbial communities may inhibit growth and antibiotic resistance evolution of a focal bacterium, in this case E. coli. This study is important for three main reasons:

(i) It is one of very few studies to provide experimental evidence that presence of the gastrointestinal microbiota changes dynamics of antibiotic resistance evolution, emphasizing the role of community interactions for evolutionary change
(ii) It is the presence of both the microbiota and an antibiotic that enhances E. coli extinction and reduces antibiotic resistance evolution, while E. coli is able to establish itself in the microbiota communities in the absence of antibiotics and to evolve antibiotic resistance in the absence of the microbiota.
(iii) Resistance evolution was not mediated by horizontal gene transfer (HGT), even though resistance-encoding mobile elements were present in the microbiota community, highlighting that HGT may be limited by genetic and environmental constraints and generally represents a rare event.

The methods are well chosen and developed; the results are reliable and have been very well analysed statistically. The study is in principle suitable for publication in Plos Biol. However, several major and minor problems still need to be improved.

Major comments:

1) The study is important because of the novel experimental approach and the insights obtained (see above points (i) and (ii)). Yet, the authors should acknowledge the study’s limitations, as only one focal strain, one antibiotic and three microbiota samples were tested. Thus, broader generalizations should be avoided and the limitations briefly described in the discussion.*

We have added discussion of these limitations (lines 509-518), while also highlighting key patterns that were repeatable across samples from different human donors (lines 518-521) and therefore probably extend beyond our experiment.

*2) The above point (ii) should be highlighted more clearly in the discussion. It is mentioned in the discussion (lines 283-285), yet in my opinion its importance does not become sufficiently clear. The authors found that the interaction between microbiota and antibiotics enhances E. coli extinction and minimizes resistance evolution in the surviving bacteria, whereas only one of the two does not. This is important, because it could otherwise be criticized that presence of the microbiota alone drives E. coli to extinction (which is usually not the case) or that E. coli is not able under these experimental conditions to evolve antibiotic resistance (which it can in the absence of the microbiota).* 

We emphasized this point more in the discussion (Line 396-399, 446-449 and 512-515), as well as providing a more explicit breakdown of how the community and ampicillin interact in the results section (line 117-120, S1 Table).

*3) The authors should assess to what extent an inoculum effect could have influenced the results. If I understood the methods correctly, then antibiotics and E. coli are used at identical concentrations in the treatment with or without microbiota communities. If correct, then the treatment with microbiota has much higher density of bacterial cells and the effective concentration of the antibiotic per bacterial cell is diluted. In turn, the focal E. coli experienced a lower effective dose of the antibiotic. In contrast, E. coli in the treatment without the microbiota experienced a much higher effective dose of the antibiotic. If correct, then it may not be surprising that E. coli only evolved antibiotic resistance in the treatment with the higher selective pressure. Please note that ampicillin is a beta-lactam antibiotic, for which such inoculum effects have been well characterized. See for example: Udekwu et al. (2009) J. Antimicrob. Chemother. 63, 745–757; or Nicoloff et al. 2019 Nature Microbiol. 4, 504–514. One option is to measure ampicillin ICs or determine ampicillin MIC for E. coli under the different cell densities.*

We have now done a specific experiment looking at the effect of inoculum size on ampicillin inhibition of our focal strain (S8 Fig; line 451). We found an inoculum effect, in that inhibition was slightly weaker at very high cell densities. However even at high densities similar to those in our evolution experiment, there was still strong growth inhibition. Moreover, our data from the main experiment indicate inhibition by ampicillin was in fact stronger in the presence of the community, which we now explain in more detail in the results section (line 117-120; S1 Table). Our new competition data support this, showing the ancestral focal strain is strongly inhibited by ampicillin in the community+ampicillin treatment, and that high-level resistance would have been very beneficial here (337-345; S5 Fig). In the same experiment, we also included a supra-MIC concentration (see response to reviewer 2), which indicated the advantage of resistance can indeed be strengthened by ramping up the antibiotic concentration. Together these data show the lack of resistance evolution in the community treatments cannot be explained by weaker antibiotic inhibition / weaker selection for resistance.

*4) Ampicillin was used at IC90, according to the methods (Line 429). Yet, there is no reduction in cell number in the microbiota-free treatments, not even at the beginning, when resistance is unlikely to have evolved de novo and/or have spread through the population. Can the authors explain this? In the worst case, some aspect of the experimental design inhibited activity of the antibiotic.*

There is in fact significant inhibition by ampicillin in both the community and community-free treatments, including at the beginning. This was difficult to see in the previous version, particularly because of the log scale in Fig 1. To better clarify this, we added an additional table (S1 Table) showing how much ampicillin reduced population size in each treatment, which was between 69 and 89 % compared to the untreated samples. This is referred to in the main text on lines 117-120.

*5) Genome sequencing data: The sequence data is not publicly available. Moreover, some summary statistics should be provided, including number of contigs, contig lengths, etc. Moreover, it is unclear whether duplications have contributed to resistance evolution. The duplication of genomic regions with resistance genes is well known to contribute to fast resistance emergence in E. coli. Therefore, this must be evaluated.*

Sequence data is now publicly available and we have added new supplementary Tables 6A+B and 7, providing summary statistics of the *de novo* assemblies and a list of all sequenced isolates. We have now also screened for duplications in the genome by looking for increased coverage above the average. We did this by determining first the average coverage of each sequenced isolate and then screened for regions that had at least twice as many reads and were longer than 500 bp, but none of the chromosomal regions fulfilled these requirements.

*6) Plasmids: Without third-generation sequencing, it is difficult to assess whether the contig of interest on human donor 3 is a putative plasmid or not. It may as well be a plasmid integrated in a genomic island, and which has lost the ability to be self-transferred. Long-read sequencing would also be helpful to fully resolved the putative plasmid from human donor 1. Moreover, it does not become clear how the absence of other plasmids was excluded (see lines 239 following).*

We have now used long-read sequencing as suggested (MinION platform) to resolve the genome architecture of the two representative isolates of resident *E. coli* from human donors 1 and 3 (Fig 4; Lines 676-679 in Methods; lines 288-295 in Results). This allowed us to close the chromosome and two plasmids for the isolate from human donor 1. For human donor 3 we were not able to completely close the chromosome, but the new long-read data did allow us to identify all the resistance genes and the plasmid replicon integrated on the chromosome. We have also clarified in the Methods that we searched for known plasmid-associated sequences using Plasmidfinder, a repository of whole-plasmid sequences from members of the *Enterobacteriaceae* (Line 682).

*Minor comments:

1) Lines 33-35: I found the final conclusions of the abstract difficult to read. I suggest simplifying them to make them more accessible to the average reader.*

We have revised this text (Line 35).

*2) The authors should briefly explain why ampicillin was selected for this study.*

We explained the rationale for using ampicillin in more detail in the introduction (Lines 87-90)

*3) Line 52: Public goods sharing could be mentioned here.*

We added a reference to public good sharing in the introduction (Line 52)

*4) Line 85: ‘chromosomally’ should be changed to ‘through new mutations‘. Please note that the beta-lactamase-encoding gene may be acquired horizontally and then be integrated in the chromosome.*

We changed 'chromosomally' to 'chromosomal mutations' (Line 86-87).

*5) Lines 93, 117 and 133: replace ‘antibiotics‘ by ‘ampicillin’ or at least use the term in singular (only one antibiotic was used).*

We replaced the term 'antibiotics' with 'ampicillin' and 'antibiotic' throughout the manuscript.

*6) Line 97: ‘multiple’ is misleading and should be replaced by ‘three’ or ’several’.*

We have replaced 'multiple' with 'three' here.

*7) Line 151: 16S should be written with a Capital S. This also applies elsewhere in the text or figures.*

We changed this throughout.

*8) Line 152 and Figure 3: I find Supp. Fig. 2 more informative than current Fig. 3. I would at least add Supp. Fig. 2 as a sub-panel of Fig. 3 to the main text.*

As suggested, we have now combined Supp. Fig. 2 with Fig 3.

*9) Lines 216 following: It would help the reader if information on the number of strains sequenced is provided here. Similarly, it would be useful to know how presence of plasmid genes was inferred.*

We provided an additional table with this information (S6+S7 Table) and clarified how we detected plasmids (Line 682).

*10) Lines 226-228: The fact that the reads map against the single contig does not mean that these isolates share the same exact plasmid, as genomic rearrangements may have occurred. Please rephrase this.*

We rephrased this in the revised section that now incorporates long-read sequence data (299-303).

*11) Lines 250 following: It would help the reader if more precise information on MICs of the various strains would be provided, e.g. in a table.*

We added an additional table (S5 Table) to the supplementary material where we provide IC90 estimates inferred from these data for each isolate.

*12) Lines 287-290: The focal strain may have CRISPR-Cas or other defense mechanisms that prevent the invasion by foreign DNA, which may help to explain the troubles the authors found in transferring the plasmid by conjugation. Since the authors sequenced the strains, these mechanisms should be explored.*

If this were the case, we would have expected such defence mechanisms to also prevent transfer on agar surfaces, which we did not observe (Fig 5B). We have nevertheless now also screened the genome of our focal strain with the CRISPRCasFinder (https://crisprcas.i2bc.paris-saclay.fr) for the presence of any spacers that match the plasmid of human donor 1. We detected 18 spacers, but none of them had sequences homologous to the plasmid.

*13) Line 312-313: The quoted papers are actually not that recent anymore. Therefore, this term should be replaced.*

We have revised the text here (line 439).

*14) Lines 324-326: The authors do not provide any experimental evidence for the conclusion drawn. Therefore, the statement should be changed or the data provided.*

We have revised the text here (Line 428) and we provide additional experimental evidence for this conclusion (S5 Fig; Line 337-345).

*15) Lines 455-456: The authors should explain the criteria used to design the primers. A quick blastn search revealed that the sequences have perfect hits with several E. coli sequences deposited in NCBI.*

*E. coli* MG1655 K12 is one of the most widely used model organisms and therefore very well represented in the NCBI database, which might explain many of the perfect hits. We designed the exclusive primers by first mapping the reads of the resident *E. coli* isolates to our focal strain. We then selected a region where no reads aligned with the focal strain reference and designed primers for this locus. We tested these primers by PCR and qPCR on the faecal slurry without any focal strain and did not get any product, whereas the focal strain did produce a product (S3+S4 Fig).

*16) Line 462: ‘SYBR Safe’.*

Revised.

*17) Lines 507, 512, 518-521, 605/612: Please mention the software/tool versions.*

We have added this information (Line 663, 668, 675, 681-682, 768-771,).

*18) Line 500: Were all the of the randomly selected strains susceptible to ampicillin? This should have been tested and the data provided.*

We have now tested this and added it in a new column of S7 Table.

*19) Line 512: ‘Ismapper’.*

We have revised this to 'ISMapper', consistent with the publication describing this tool.

*20) Line 556: This explanation should be moved to the first time Resazurin is mentioned (line 385).*

We moved this explanation further up (Line 551).

*21) Lines 586-589: Please rephrase this sentence.*

We have revised the text here (747-750).

*22) Lines 588-589: Please provide names of the extraction kits.*

We added the name of the extraction kit (line 751).

*23) Figure 1. Please provide information on which initial time points are shown. This cannot be inferred from the graphs.*

We revised the figure legend to clarify that the first time points are 0h and 2h, with ampicillin being applied after 2h.

*24) Line 938 and Fig. 4 A and B: TEM in blaTEM should be subscript.*

We have corrected this in the text.
